# Statistical Analysis for Subjective and Objective Evaluations of Dental Drill Sounds

**DOI:** 10.1371/journal.pone.0159926

**Published:** 2016-07-27

**Authors:** Tomomi Yamada, Sonoko Kuwano, Shigeyuki Ebisu, Mikako Hayashi

**Affiliations:** 1 Department of Restorative Dentistry and Endodontology, Osaka University Graduate School of Dentistry, Suita, Osaka, Japan; 2 Department of Human Science, Osaka University Graduate School of Human Science, Suita, Osaka, Japan; University of California, Irvine, UNITED STATES

## Abstract

The sound produced by a dental air turbine handpiece (dental drill) can markedly influence the sound environment in a dental clinic. Indeed, many patients report that the sound of a dental drill elicits an unpleasant feeling. Although several manufacturers have attempted to reduce the sound pressure levels produced by dental drills during idling based on ISO 14457, the sound emitted by such drills under active drilling conditions may negatively influence the dental clinic sound environment. The physical metrics related to the unpleasant impressions associated with dental drill sounds have not been determined. In the present study, psychological measurements of dental drill sounds were conducted with the aim of facilitating improvement of the sound environment at dental clinics. Specifically, we examined the impressions elicited by the sounds of 12 types of dental drills in idling and drilling conditions using a semantic differential. The analysis revealed that the impressions of dental drill sounds varied considerably between idling and drilling conditions and among the examined drills. This finding suggests that measuring the sound of a dental drill in idling conditions alone may be insufficient for evaluating the effects of the sound. We related the results of the psychological evaluations to those of measurements of the physical metrics of equivalent continuous A-weighted sound pressure levels (*L*_Aeq_) and sharpness. Factor analysis indicated that impressions of the dental drill sounds consisted of two factors: “metallic and unpleasant” and “powerful”. *L*_Aeq_ had a strong relationship with “powerful impression”, calculated sharpness was positively related to “metallic impression”, and “unpleasant impression” was predicted by the combination of both *L*_Aeq_ and calculated sharpness. The present analyses indicate that, in addition to a reduction in sound pressure level, refining the frequency components of dental drill sounds is important for creating a comfortable sound environment in dental clinics.

## Introduction

The sound that is most often associated with dental treatment is that of a dental air turbine handpiece (dental drill). The sound emitted by dental drills can have a powerful influence on the sound environment in a dental clinic. Indeed, a questionnaire that surveyed patients regarding their impression of dental situations found that approximately half of the respondents experienced an unpleasant feeling when they heard drilling sounds related to dental treatment [1]. The Occupational Safety and Health Administration mandates that employers provide hearing conservation programs for their employees in workplaces where noise levels are equal to or exceed 85 dB for the eight hour equivalent continuous A-weighted sound pressure level in dB, referenced to 20 micropascals (*L*_Aeq_, _8 h_) [2]. Several reports have measured the noise level at dental clinics and have found that the noise levels to which dentists are exposed is below the limit for risk of hearing damage [3, 4]. Although the A-weighted sound pressure level generated by dental drills has been standardized so that it is not expected to exceed 80 dB during idling operation (ISO 14457 [5], JIS T 5906 [6]), further efforts to create a comfortable sound environment in dental care settings are warranted.

Recently, the sound quality of various sounds has been examined to create more pleasant sounds or specific sounds such as warning signals that provide helpful information [7–16]. Generally, the main target for improving noise from various sources, particularly machines, is the reduction of sound pressure levels; however, technical and economic concerns often limit the capacity to reduce sound pressure levels. Various psychological measurements have been used to examine the relationship between physical values and subjective judgment in noise research and product sound quality engineering. The Semantic Differential proposed by Osgood [17] is often used to measure sound quality. This method is a scaling technique for measuring the connotative meaning of certain verbal concepts and is based on sets of antonym/adjective pairs, such as “good-bad” and “weak-strong". Using this approach, researchers and engineers have attempted to design optimal sounds for vehicles, machines, and other consumer products, and to create pleasant sound environments in offices, public transport stations, airports and highways. To our knowledge, however, psychological measurements related to dental drill sounds have not been previously reported. An assessment of the physical values associated with the subjective impressions of such sounds may enable modification of the sound of dental drills, thus improving the sound environment at dental clinics. In our previous study [1], those who answered that the sound of a dental drill was unpleasant tended to be more fearful of dental treatment than the other respondents and the fear of the sound of a dental drill had a strong influence on dental anxiety level. In particular, half of those who answered that they were very fearful had the experience of avoiding a dental treatment. A comfortable sound environment at dental clinics would likely encourage people to visit more frequently for dental evaluations and treatment, and may facilitate the maintenance of dental health, and accordingly, quality of life.

In the present study, psychological evaluation of the subjective impression of dental drill sounds and measurements of the physical properties of sounds emitted by dental drills in both idling and drilling conditions were conducted and examined the relationships between the subjective impression and physical values to find the physical metrics of unpleasant dental drill sounds.

## Methods

All experiments were approved by the Ethics Committee of the Osaka University Graduate School of Dentistry. All participants provided written informed consent before taking part in the experiments.

### Preliminary experiment for selection of adjective pairs

As a preliminary experiment, we collected information on the subjective impressions of dental drill sounds from 191 females ranging in age from 18 to 24 years. Using the resulting data, suitable antonym/adjective pairs were selected for psychologically evaluating the impact of the sound of dental drills on the sound environment in dental clinics. The participants were freshmen in a dental hygienist course and the survey was conducted before they had received practical dental training. Each participant was asked to complete a questionnaire in which they evaluated their impressions of the sounds of dental drills by ranking a list of 11 adjectives on a 5-point scale. The participants were also asked to provide any adjectives or comments that they felt appropriate to express their impression of the sound of dental drills.

**Fig 1:**
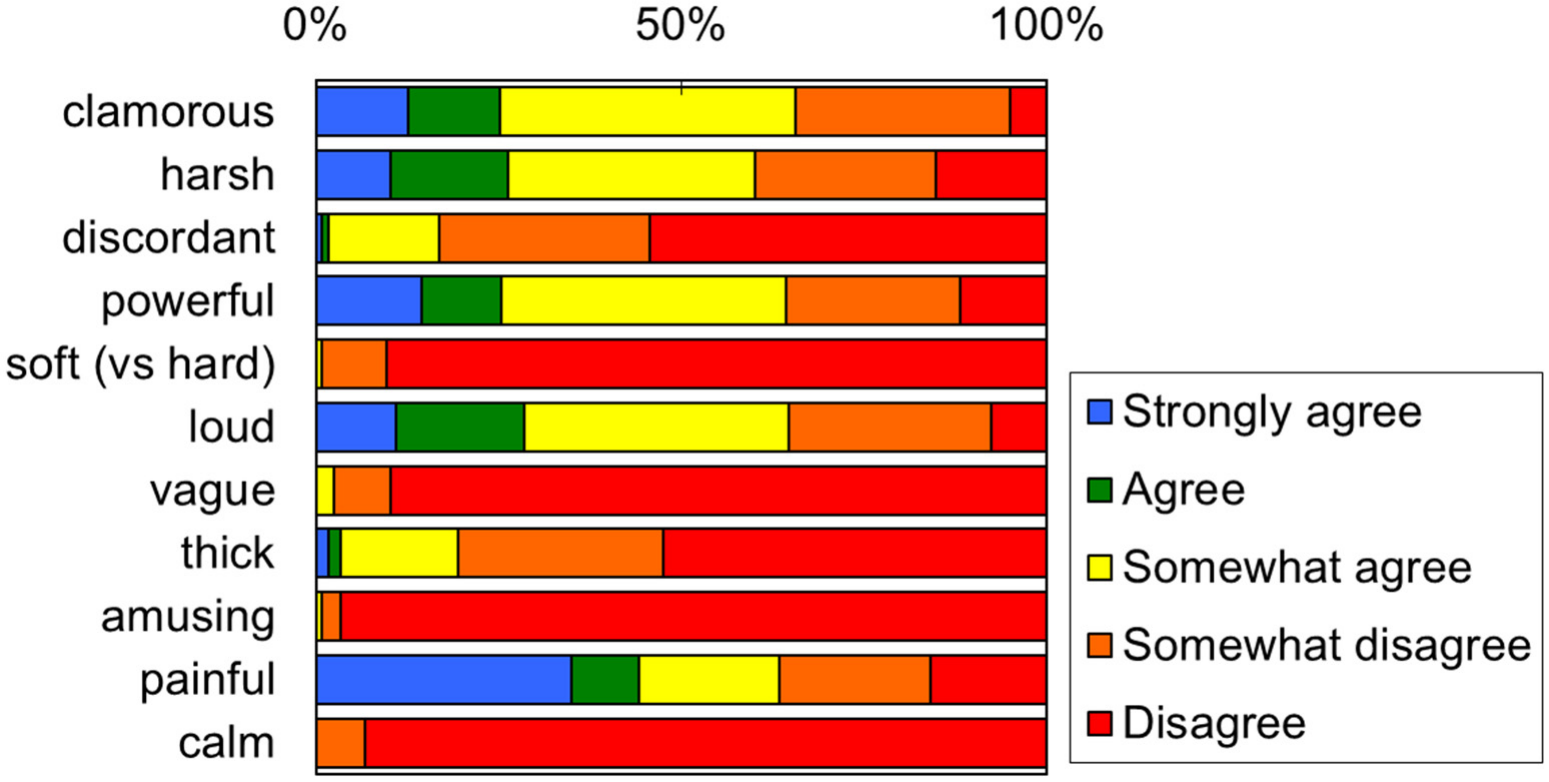
Participant answers to the question: “How much do you agree that each adjective is appropriate to express the impression of the sound of dental drills?” Participants were asked to score their responses for each adjective on a 5-point scale ranging from “strongly agree” (level 1) to “disagree” (level 5).

The results of the preliminary experiment are shown in Fig 1. An evaluation of the participant responses to the 11 adjective rating scales revealed that approximately 60% of the participants felt the sounds of dental drills were “clamorous”, “harsh”, “powerful”, “loud” and “painful”. In addition to the 11 prepared adjectives, the participants provided an additional 142 adjectives to describe the sound of dental drills. Most of the adjectives described characteristics of the sound, such as “high-pitched tone”, “sharp” and “metallic” (n = 29), or negative emotions, such as “frightened”, “scary”/“fearful” (n = 33), “dislike” (n = 20), “tense” (n = 8), and “unpleasant”/“unpleasing” (n = 7). Several participants even stated that the sound of dental drills “makes me feel sick (n = 2)”, elicits the feeling that “I want to run away (n = 2)” or causes a specific ailment, “I get a headache (n = 4)/ toothache (n = 3)/ earache (n = 9)”.

Based on the results of the preliminary experiment, we chose 15 adjective pairs for the psychological evaluation of dental drill sounds. Among the 15 adjective pairs selected, the adjectives “metallic”, “harsh”, “sharp”,”shrill”, “hard”, and “distinct” represented the characteristics of the sound. The adjectives “unpleasant”, “dislike”, “tense”, “unpleasing”, “painful”, and “fearful” represented the emotions associated with the sound, and the adjectives “loud”, “powerful”, and “clamorous” represented the power of the sound.

### Psychological experiment

#### Sound stimuli

The list of 12 dental drills used is shown in Table 1. The sounds of the dental drills were recorded under both idling and drilling conditions at Osaka University Dental Hospital in a quiet environment. To simulate drilling conditions in a clinical setting, an artificial tooth (A20-500; Nisshin) with two hardness layers (enamel and dentin) was drilled to a standardized depth for dental fillings by an experienced dentist. Each dental drill was operated using the air pressure level recommended by the corresponding manufacturer. During idling and drilling conditions, a water/air spray mixture from the head of the dental drill was used to cool the frictional heat generated by drilling. The sounds emitted by the dental drills were recorded using a 1/4 inch-diameter condenser microphone (UC-29; Rion), a sound level meter (NA-40; Rion), and a DAT recorder (DTC-ZA5ES; Sony). The microphone was positioned 30 cm from the drill head, as this was the closest position for recording that minimized the amount of water that contacted the microphone. Recording sounds of dental drills were edited to a length of approximately 5 s each using sound software (Audition 2.0; Adobe). The labels of the stimuli, A—L, do not always correspond to the order in the list of the dental drills in Table 1. In total, 24 sound stimuli were prepared.

**Table 1:** List of dental drills used.

Model	Manufacturer
Synea HS TA-98L	W&H Dentalwerk Bürmoos GmbH, Austria
T1 CONTROL	Sirona Dental Systems GmbH, Germany
T2 Racer	Sirona Dental Systems GmbH, Germany
GENTLE force LUX 7000B	Kaltenbach & Voigt GmbH, Germany
Super Torque LUX3 650B	Kaltenbach & Voigt GmbH, Germany
Super Torque LUX3 640B	Kaltenbach & Voigt GmbH, Germany
ASTRON SUPER α2	J. MORITA MFG. CORP., Japan
ASTRON SUPER ZA	J. MORITA MFG. CORP., Japan
JETMASTER STα	J. MORITA MFG. CORP., Japan
TWINPOWER TURBINE p	J. MORITA MFG. CORP., Japan
TWINPOWER TURBINE 4H	J. MORITA MFG. CORP., Japan
Ti-Max A600L	Nakanishi Inc., Japan

#### Procedure

We conducted psychological measurements using the semantic differential scale. The sound stimuli were produced by 12 types of dental drills, recorded in both idling and drilling conditions. During the experiment, participants were seated alone in a sound-proof room at the Graduate School of Human Sciences, Osaka University, and instructed to judge the impression of the sound stimuli delivered through headphones (SRM-313; STAX) using the 15 adjective pairs selected on the basis of the preliminary experimental results. Before exposure to the test stimuli, participants received training trial regarding the experimental procedure using two sounds that were not included in the experiment. The participants were informed that the test stimuli consisted of the recordings of dental drill sounds. Participants were asked to judge their impressions of 24 sound stimuli twice in two separate sessions. The participants repeated the experiment after a period of rest (approximately 30 min) or on a different day. Subjective impressions of the stimuli were evaluated using semantic differential scales. To minimize the potential influence of the adjective order on the evaluations, we prepared three kinds of adjective lists in a different order, and participants evaluated their impressions of the sound stimuli using one of the three lists.

#### Participants

Three females and eighteen males aged between 21 and 29 years (average 23.2 years) with normal hearing ability participated in this experiment. All of the participants were Japanese and had experienced dental treatment, and none were dental students.

### Objective metrics

#### Physical properties of sound stimuli

Among numerous possible metrics [18–24], we measured three representative physical values of sound quality: calculated loudness [19], equivalent continuous A-weighted sound pressure level (in dB) referenced to 20 micropascals (*L*_Aeq_) [20], and calculated sharpness [21]. Loudness of a sound (sone) is the primary psychological correlate of intensity of the sound. Specifically, it is a perceptual measure of the effect of the energy content of the sound on the ear [19]. *L*_Aeq_ (dB) is an energy-based noise metric that is widely used to evaluate temporally varying sounds and was adopted as a basic measure of environment noise in ISO 1996, Part 1 [20]. Calculated sharpness (acum) has been used to partially quantify sound quality and is considered to be an attribute of timbre. Von Bismarck [21] introduced the concept of a weighted first moment calculation for sharpness. Fastl and Zwicker [18] defined a sound with a ‘narrow band noise of one critical band width at a center frequency of 1 kHz having a level of 60 dB’ as 1 acum sharpness. The stimuli were reproduced with headphones (SRM-313; STAX) and were recorded for physical analyses using a Head and Torso Simulator (HATS; Brüel and Kjær), which is a manikin with built-in ear simulators. *L*_Aeq_ values were measured using a sound level meter (LA1250; Ono Sokki). Loudness and sharpness were calculated using sound quality software (7698, 5265; Brüel and Kjær). The software adopted the methods of Fastl and Zwicker for using a modified weighting curve in Von Bismarck’s equation to calculate sharpness.

#### Comfort Index

Although various sound quality indices have been proposed [8, 21, 23, 25, 26], no metrics exist that can be widely applied to all sounds. For this reason, Kuwano et al. proposed a new index, termed the Comfort Index (*CI*), at the Seminar on The Sound Quality Study and it’s Application to Traffic Noise (2003). The *CI* [12] consists of *L*_Aeq_ and sharpness, and is defined by the following equation:

$$CI=\;1/10L_{Aeq}+\text{ sharpness}$$ (1)

In this equation, *L*_Aeq_ can be replaced with *LL*z, which is a mean energy level to apply ISO 532 B to temporally varying sounds [27, 28]. *CI* was shown to have a good correlation with the sound quality of traffic noises and copy machine noise [12, 29].

We examined the applicability of *CI* to sound emitted by dental drills in this study.

### Statistical analysis

All statistical analyses were performed using SPSS statistical software (SPSS, Inc.). A p value of <0.01 was considered statistically significant.

## Results and Discussion

### Subjective evaluation

#### Reliability of the psychological evaluation

We found a significant correlation between the results from the two psychological evaluation sessions in all participants (Spearman’s rank correlation, correlation coefficient r = 0.86, p<0.01). This result indicates that the judgment of all participants was reliable. Therefore, we combined the results of the two trials of the psychological evaluations in the following analyses.

#### Subjective impressions to sounds of dental drill

**Fig 2:**
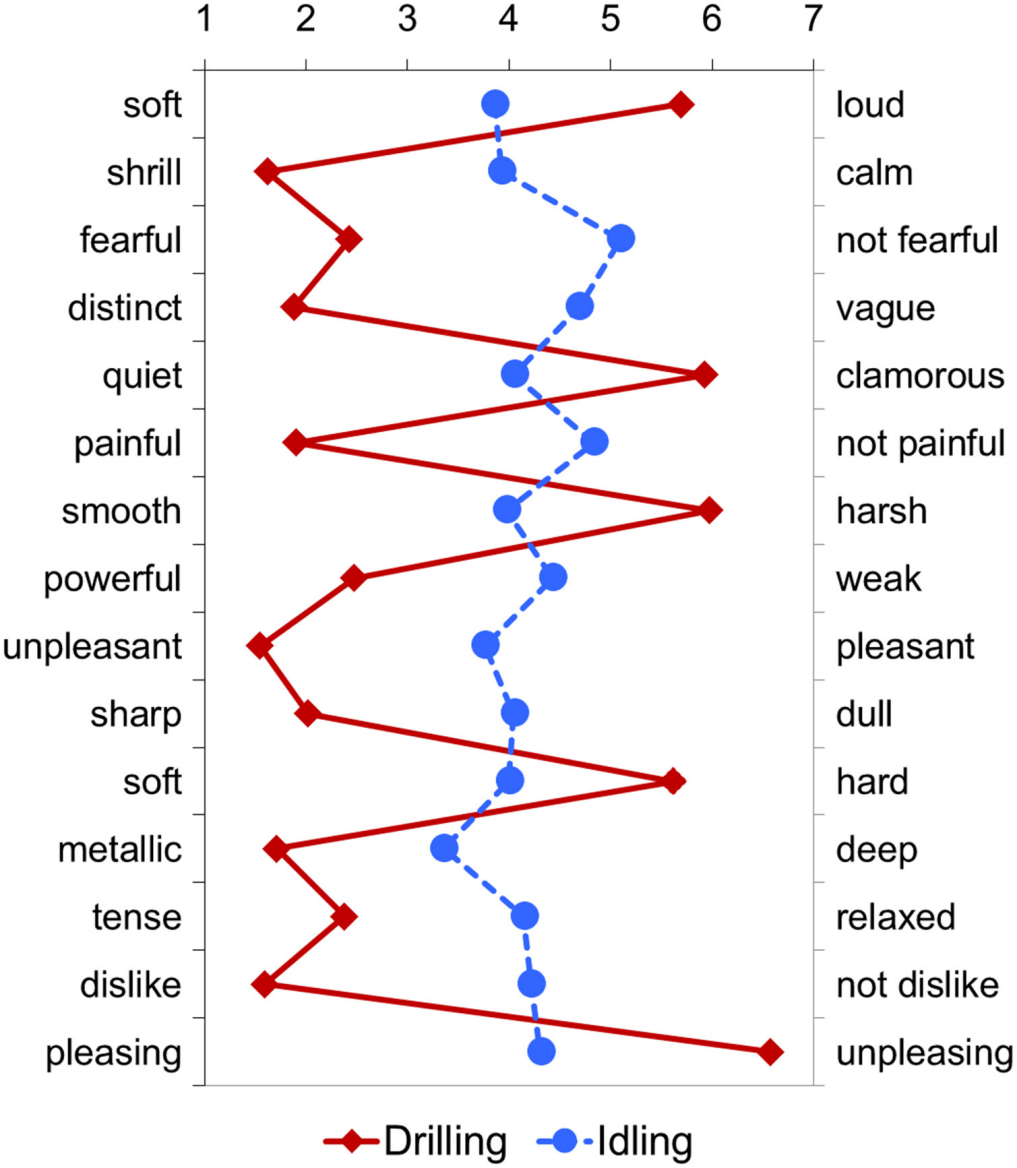
Subjective impressions to the sounds of Drill E.

We observed a marked difference between the subjective impression to sounds emitted under idling and drilling conditions. For Drill E, we observed a marked difference in the subjective impressions to the sound emitted under idling and active drilling conditions (Fig 2). Based on the values for the 15 adjective pairs, the sound of Drill E under idling conditions was judged to be the most favorable among the 12 drills. However, the sound of Drill E under active drilling conditions was perceived to be the most unfavorable as found on the adjective scales, “shrill”, “unpleasant”, and “dislike”.

We also found differences in “unpleasant impression” of the sounds emitted by most dental drills under idling and drilling conditions. In the value distribution for the “unpleasant impression” for the stimuli under drilling conditions, nearly all of the examined sound stimuli were judged to be highly “unpleasant”. Here, none of the stimuli emitted by the dental drills under drilling condition were perceived by participants as satisfying.

### Objective evaluation

#### Comparison of the physical properties of dental drill sounds between idling and drilling conditions

**Fig 3:**
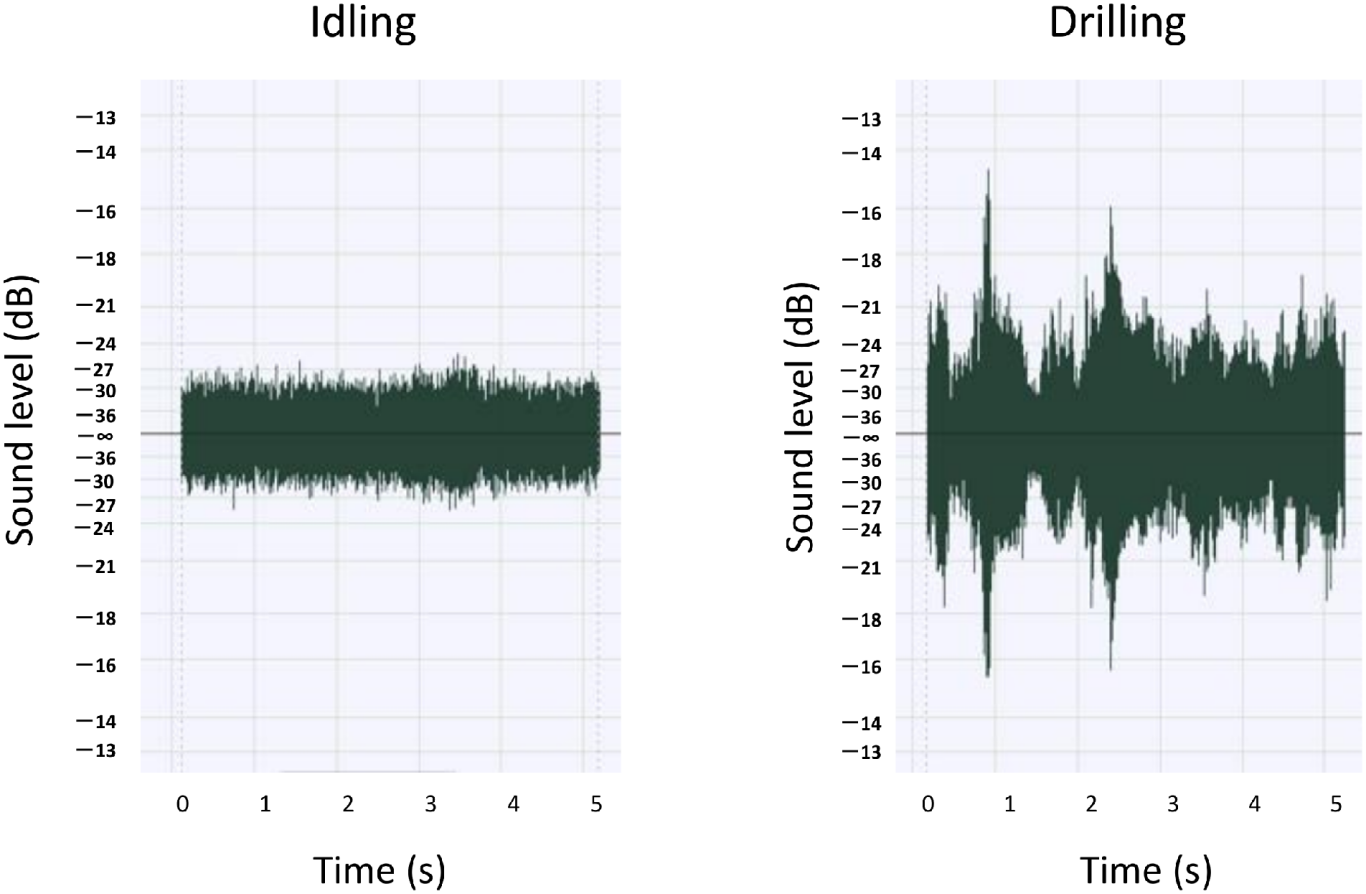
Waveforms of Drill E under idling and drilling conditions.

**Fig 4:**
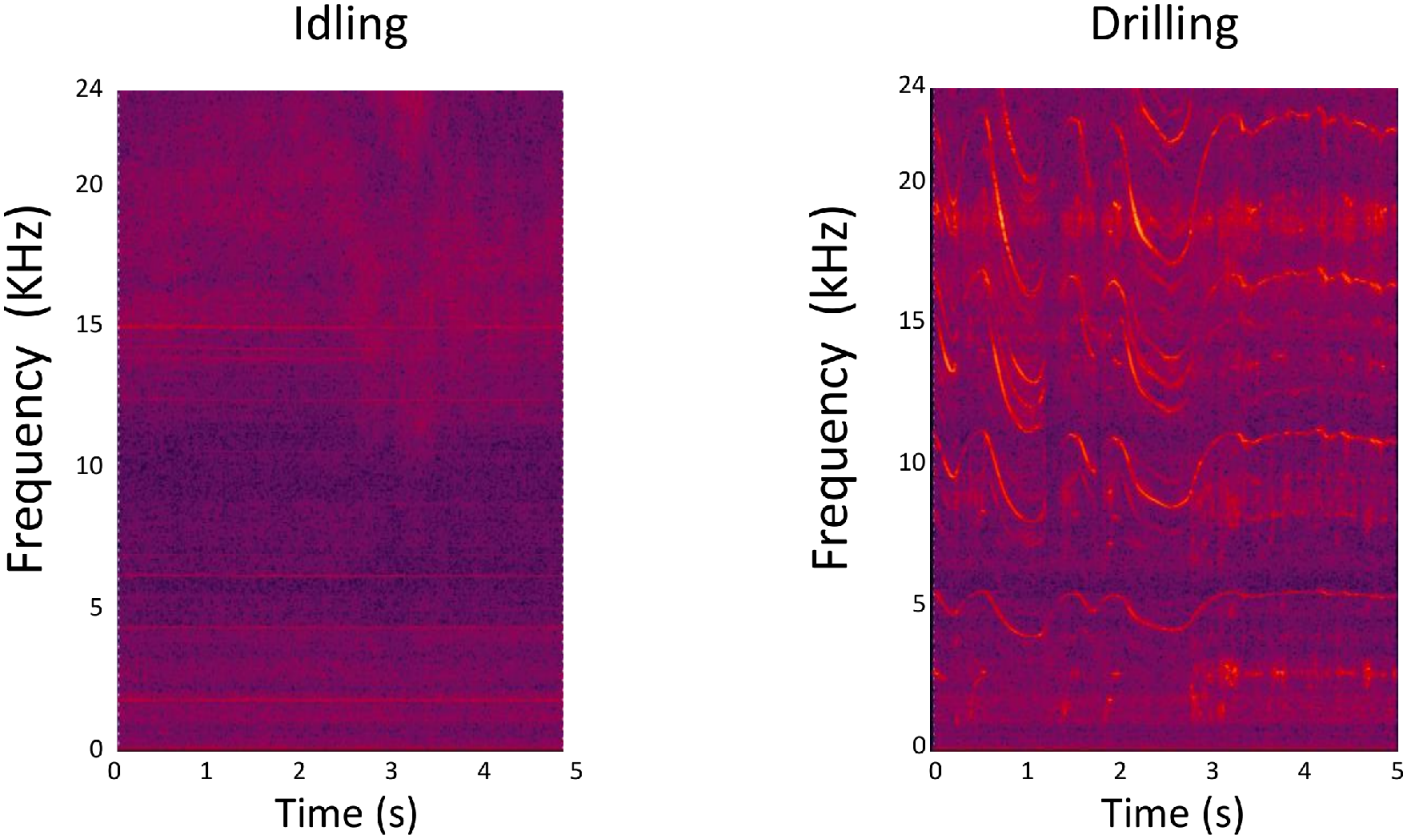
Spectrograms of Drill E sound. A spectrogram is an image that incorporates frequency (y axis), time (x axis), and amplitude (brightness of color).

We measured the physical properties and also examined the fluctuation of the sound levels emitted by the 12 dental drills under idling and drilling conditions. Under idling conditions, all dental drills emitted a relatively stable sound, whereas drilling of an artificial tooth produced a fluctuating sound. For example, a large difference in the sound level between idling and drilling conditions was detected for Drill E (Fig 3). In addition, greater fluctuation in the sound pressure level (no frequency weighting) was typically observed under drilling conditions compared with idling conditions. Fig 4 shows the spectrograms of Drill E sound. Spectra of sound of dental drills under operational condition had several prominent frequency components in the wide frequency region and were different among the dental drills.

The values for *L*_Aeq_, calculated loudness, and sharpness of the sound stimuli for the 12 drills are shown in Table 2. We detected differences in the physical properties of the sounds among the 12 dental drills and between the operating conditions. The *L*_Aeq_ values obtained under idling conditions ranged from 65.9 dB (Drill L) to 76.6 dB (Drills B and C), whereas the *L*_Aeq_ values between drilling and idling conditions ranged from 0.5 dB (Drill C) to 6.6 dB (Drill L). The calculated loudness values under idling conditions ranged from 12.6 sone (Drill A) to 19.1 sone (Drill C), whereas the loudness values between drilling and idling conditions ranged from 0.2 sone (Drill G) to 3.2 sone (Drill A). Sharpness values under idling conditions ranged from 2.7 acum (Drill A) to 4.0 acum (Drill G), whereas the sharpness values between drilling and idling conditions ranged from 0.1 acum (Drill F) to 1.1 acum (Drill E).

**Table 2:** Physical properties of the sounds produced by 12 types of dental drills.

Drill	*L*_Aeq_ (dB)		Loudness (sone)		Sharpness (acum)	
	Idling	Drilling	Idling	Drilling	Idling	Drilling
A	74.1	75.8	12.6	15.8	2.7	3.2
B	76.6	77.5	17.1	15.6	3.2	3.7
C	76.6	77.1	19.1	20.7	3.3	3.5
D	70.4	70.9	15.8	17.3	3.6	4.0
E	69.3	73.0	13.5	15.7	3.0	4.1
F	68.5	72.4	14.5	16.3	3.8	3.9
G	67.7	68.4	13.9	14.1	4.0	4.2
H	68.3	67.3	12.8	14.1	3.4	4.0
I	69.0	69.6	12.8	14.4	3.4	3.7
J	67.5	70.4	13.2	14.3	3.8	3.5
K	70.6	71.6	14.7	15.7	3.5	3.6
L	65.9	72.5	14.0	17.0	3.7	4.1

We also detected a significant correlation between *L*_Aeq_ and calculated loudness (Spearman’s rank correlation, r = 0.84). In contrast to loudness, no correlation between *L*_Aeq_ and calculated sharpness was found. For this reason, we conducted the following analyses using *L*_Aeq_ and calculated sharpness to evaluate the relationship between physical values and the subjective impressions of dental drill sounds.

### Statistical analysis

#### Differences in subjective impressions among dental drill sounds

To examine the different characteristics among the sound stimuli, we conducted a cluster analysis. The analysis revealed that participants’ impressions to dental drill sounds could be divided into two groups (p<0.01). One cluster included the sounds of 10 drills in the idling condition, and the other cluster comprised the sounds of all 12 drills under drilling conditions, as well as the idling sounds of Drills B and C. The inclusion of these idling sounds in the drilling group was likely due to the fact that the *L*_Aeq_ and loudness values of both Drills B and C were markedly higher than those of the other examined drills (Table 2). Both stimuli were judged as being louder than the idling sounds of the other drills. We also examined the distribution of judgment for each adjective and all stimuli to detect differences between the groups. The results of the cluster analysis indicated that the sounds emitted by the drills under drilling conditions were evaluated as more “clamorous”, “unpleasant”, and “sharp” than those emitted by dental drills under idling conditions. ISO 14457 has standardized the method of testing the noise level of dental drills [5]. The method uses a non-rigid suspension system and is therefore only capable of measuring sound emitted under idling conditions. However, the present findings suggest that evaluations of dental drill sound should be performed under both idling and drilling conditions.

#### Factors associated with the subjective impression of dental drill sounds

Next we conducted factor analysis [30] using the Varimax rotation method to identify the factors that composed the subjective impressions to dental drill sounds. As shown in Table 3, two factors were extracted. Factor 1 consisted of the adjectives “metallic”, “harsh”, “sharp”,”shrill”, “hard”, “distinct”, “unpleasant”, “dislike”, “tense”, “unpleasing”, “painful”, and “fearful”, and Factor 2 consisted of the adjectives “loud”, “powerful”, “clamorous”, “fearful”, “dislike”, “unpleasing”, “painful”, “unpleasant”, “distinct”, and “tense”. The cumulative variance was 61.6%, of which Factor 1 comprised 38.3% and Factor 2 comprised 23.3%. These results suggest that the majority of participants’ impressions of the sounds of dental drills consisted of two factors: “metallic and unpleasant” and “powerful”.

**Table 3:** Results of factor analysis for the adjective pairs used to characterize dental drill sounds.

Adjective pair	Factor 1	Factor 2
metallic-deep	0.786	0.234
smooth-harsh	-0.756	-0.232
sharp-dull	0.715	0.009
shrill-calm	0.693	0.278
soft-hard	-0.635	-0.329
distinct-vague	0.581	0.445
unpleasant-pleasant	0.702	0.451
dislike-not dislike	0.701	0.486
tense-relaxed	0.672	0.433
pleasing-unpleasing	-0.660	-0.477
painful-not painful	0.658	0.460
fearful-not fearful	0.524	0.537
soft-loud	-0.009	-0.747
powerful-weak	0.335	0.718
quite-clamorous	-0.339	-0.647

#### Relationship between subjective evaluation of dental drill sounds and physical metrics

We examined the relationships between the adjective scales and the physical metrics of the stimuli. The results of Spearman's correlation coefficient regarding the rank of the 15 adjective scales and the *L*_Aeq_ and sharpness values of the stimuli are presented in Table 4.

**Table 4:** Correlation coefficients between *L*_Aeq_, sharpness of the stimuli, and the 15 adjective scale values.

Adjective pair	*L*_Aeq_	Sharpness
soft-loud	0.827*	0.011
quite-clamorous	0.797*	0.017
powerful-weak	0.664*	0.153
pleasing-unpleasing	0.450*	0.367
dislike-not dislike	0.436*	0.426*
fearful-not fearful	0.427*	0.340
tense-relaxed	0.424*	0.444*
unpleasant-pleasant	0.401	0.499*
painful-not painful	0.351	0.478*
distinct-vague	0.444*	0.432*
soft-hard	0.398	0.436*
shrill-calm	0.336	0.558*
smooth-harsh	0.298	0.552*
metallic-deep	0.188	0.693*
sharp-dull	0.117	0.638*

*significance level (p<0.01)

**Fig 5:**
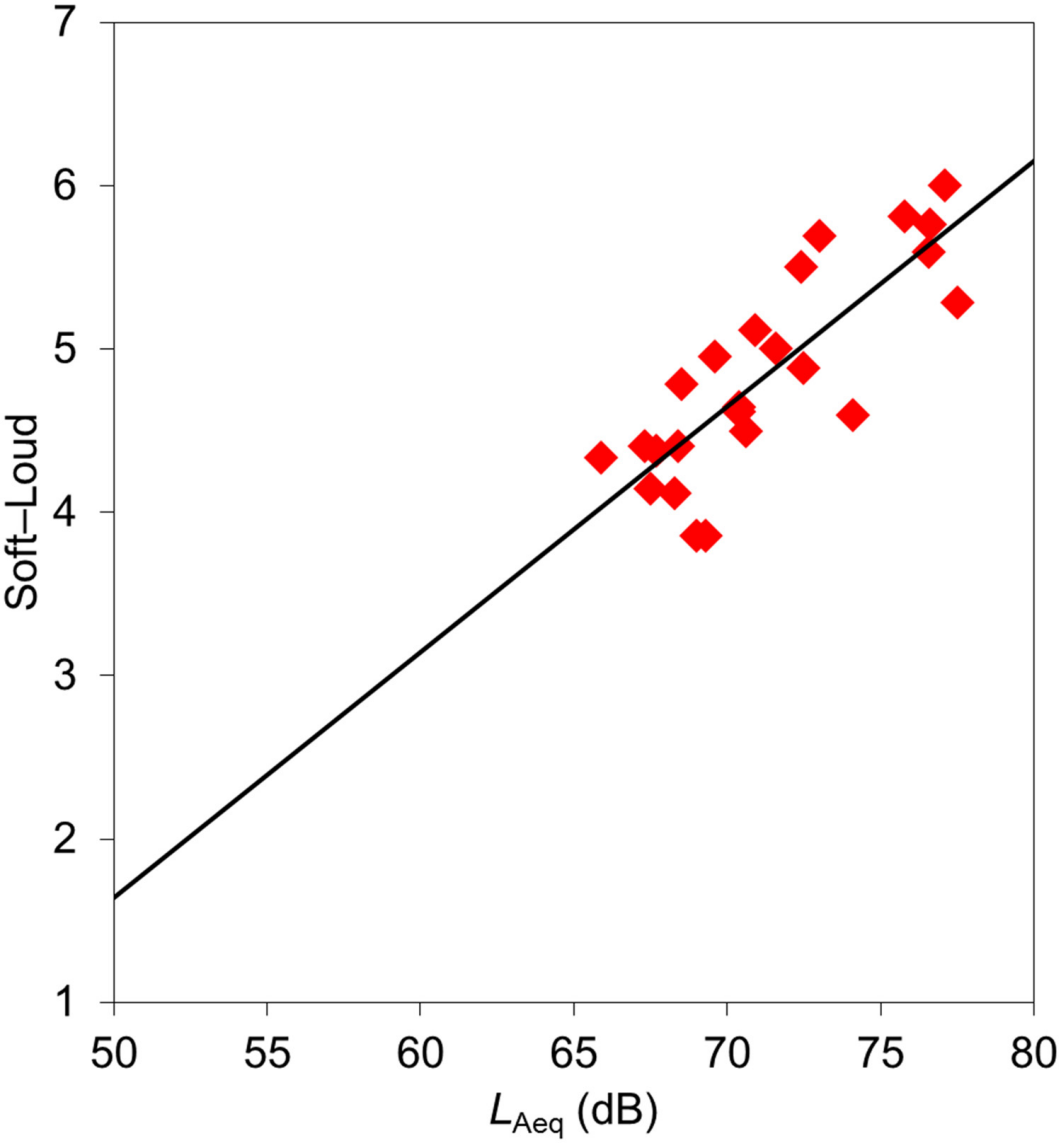
Relationships between *L*_Aeq_ values and adjective scale values for “soft-loud”.

**Fig 6:**
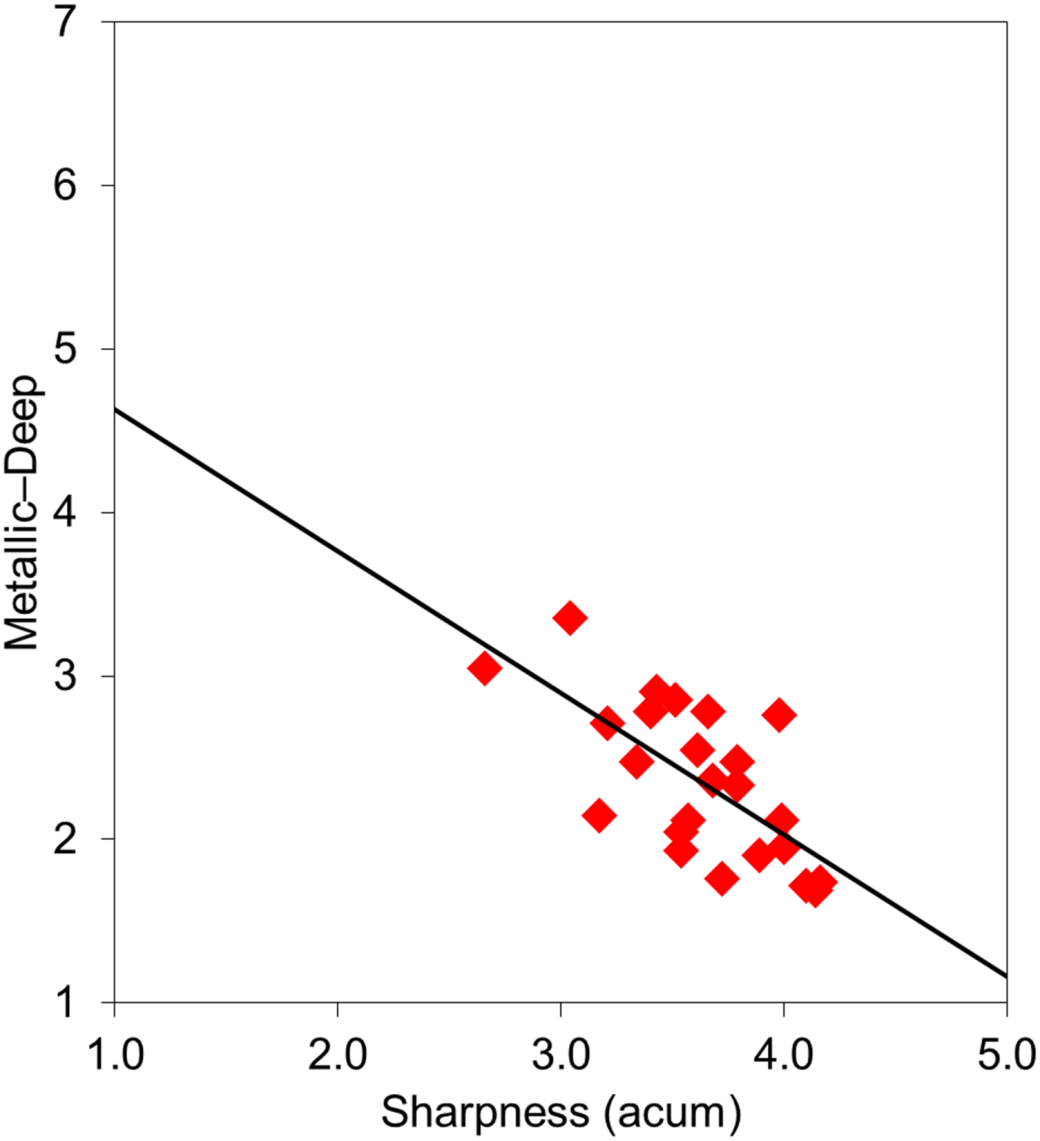
Relationships between calculated sharpness and adjective scale values for “metallic-deep”.

*L*_Aeq_ was significantly correlated with “powerful impression”, which comprised the adjective pairs “soft-loud”, “quiet-clamorous”, and “powerful-weak”. Several of the adjective scale values for “unpleasant impression”, such as “unpleasing”, “dislike”, “fearful”, and “tense”, were positively correlated with *L*_Aeq_. Calculated sharpness had an influence on “metallic impression”, which consisted of the adjectives “metallic”, “sharp”, “harsh”, “shrill”, “hard”, and “distinct”. Several of the adjective scale values for “unpleasant impression”, i.e., “dislike”, “tense”, “unpleasant”, and “painful”, were also positively correlated with calculated sharpness. Fig 5 shows the relationships between the *L*_Aeq_ values and the adjective scale values for “soft-loud”. Fig 6 presents the relationships between calculated sharpness and the adjective scale values for “metallic-deep”.

The scale values for the adjective pair “soft-loud” showed a good correlation with *L*_Aeq_ (Fig 5). We also detected a significant correlation between *L*_Aeq_ and the calculated loudness of the stimuli emitted by the dental drills. Namba and Kuwano [25] reported a good correlation between *L*_Aeq_ and the loudness impression of non-steady state sounds, as referred to in JIS Z8731 “Acoustics—Description and Measurement of Environmental Noise” [31]. Environment Quality Standard of Noise was revised in Japan and adopted *L*_Aeq_ as an index of noise criteria. The findings of the present experiment indicate that *L*_Aeq_ is appropriate for the evaluation of adjective scale values corresponding to the degree of the “powerful impression” regarding the sound of dental drills. Our analysis also revealed that calculated sharpness was significantly correlated with the adjective pair “metallic-deep” (Fig 6). This finding is consistent with studies by Kuwano at el. [16] and Namba et al. [26], who reported that sharpness and “metallic factor” were positively correlated for various sounds. Taken together, the present results indicate that sharpness is a suitable metric for evaluating the metallic impression of the sound emitted by dental drills.

Although we found a significant correlation between unpleasant impression and physical metrics, it is difficult to account for the “unpleasant impression” associated with the sound of a dental drill by examining one physical metric alone, such as *L*_Aeq_ or calculated sharpness. Thus, to examine the relationship between the unpleasant impressions and physical properties of the sounds emitted by dental drills, we performed multiple regression analyses (Table 5). The evaluation score for “unpleasing” was estimated using the following equation obtained by multiple regression analysis:

$$\text{Evaluation value of }“\text{unpleasing}”\;=\;0.62\;x\;L_{\text{Aeq}}+\;0.62\;x\text{ Sharpness}$$ (2)

**Table 5:** Results of multiple regression analysis.

Adjective pair	R	R squared	*Beta*	
			*L*_Aeq_	Sharpness
unpleasing-pleasing	0.71	0.50	0.62*	0.62*
dislike-not dislike	0.73	0.53	0.66*	0.62*
fearful-not fearful	0.67	0.45	0.61*	0.58*
tense-relaxed	0.75	0.56	0.70*	0.60*
unpleasant-pleasant	0.72	0.52	0.67*	0.59*
painful-not painful	0.70	0.49	0.53*	0.68*

*significance level (p<0.01)

This analysis revealed that a number of “unpleasant” subjective impression values with high factor loading were significantly and positively associated with *L*_Aeq_ and sharpness. In addition, the present findings suggest that a reduction of both the sound level and high frequency components related to sharpness may improve the sound quality of dental drill sounds.

#### Applicability of the Comfort Index (CI) to dental drilling sounds

**Fig 7:**
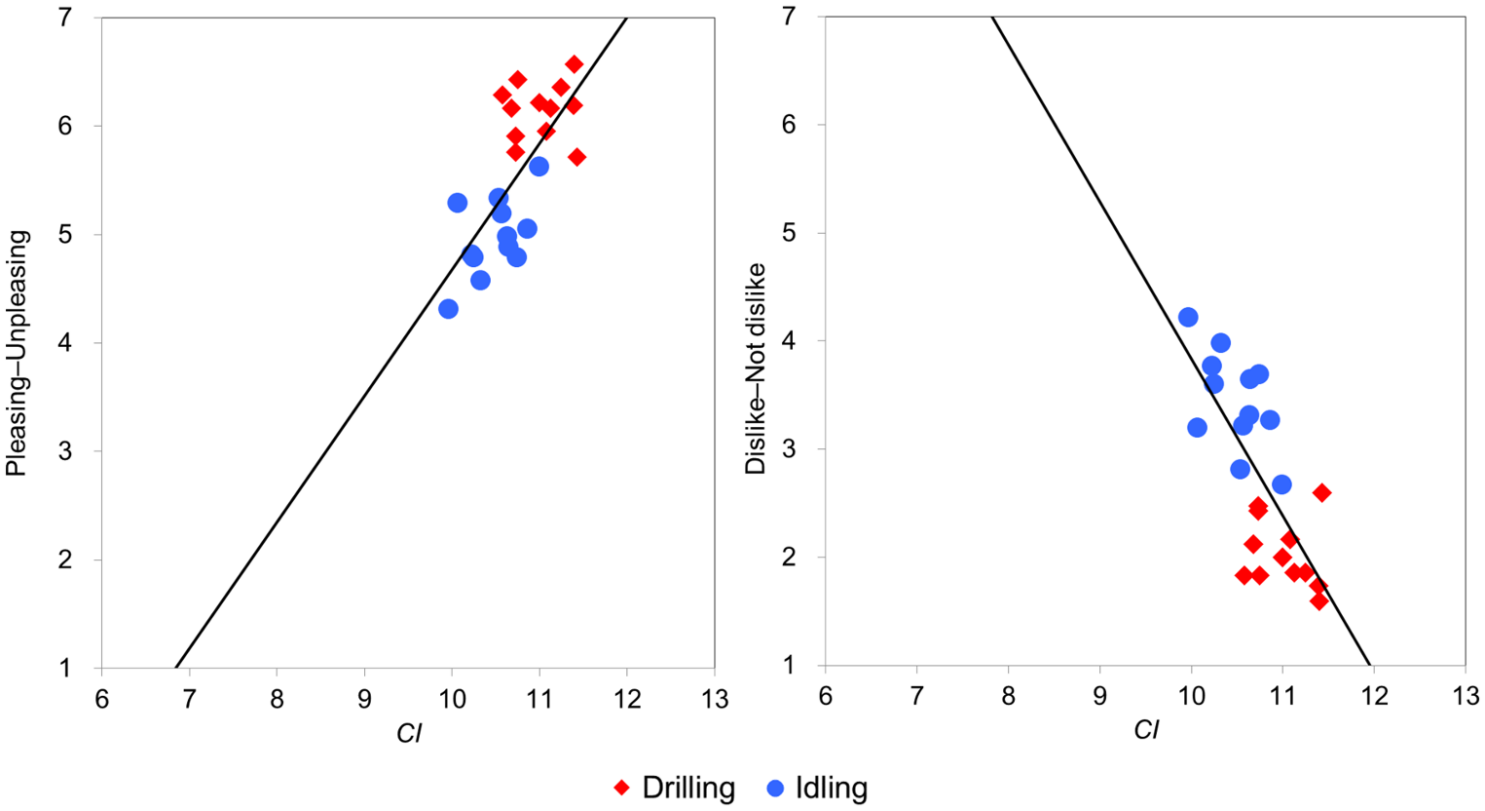
Relationship between *CI* (*L*_Aeq_/10+sharpness) and the adjective scale values ‘pleasing-unpleasing’ and ‘dislike-not dislike’.

To examine the applicability of *CI* to the sound emitted by dental drills, we extracted the correlations between *CI* and the scale values of the six adjective pairs related to “unpleasant impression” identified in the multiple regression analysis. The correlations between *CI* and all the adjective scale values listed in Table 6 were found to be statistically significant. The results of the values for “pleasing-unpleasing” and “dislike-not dislike” and *CI* are shown in Fig 7. In contrast to multiple regression analysis, which requires complicated calculations and does not guarantee the applicability of coefficients obtained by multiple regression analysis in one experiment to the data of other experiments, *CI* is easily calculated and may possibly be widely applicable. The present results indicate that *CI* is useful as a first approximation of sound quality of dental drilling sound.

**Table 6:** Correlation coefficients between adjective scale values and comfort index.

Adjective pair	Comfort Index (*CI*)
pleasing-unpleasing	0.72*
dislike-not dislike	0.73*
fearful-not fearful	0.75*
tense-relaxed	0.67*
unpleasant-pleasant	0.70*
painful-not painful	0.69*

*significance level (p<0.01)

### Dental drill sounds detected by patients

Several limitations of the present study warrant mention. First, it is possible that the sound levels of the stimuli used in the experiment were lower than those experienced clinically. The dental drill sounds were recorded with a microphone that was located 30 cm from the drill head, as this was the closest position for recording that minimized water contact with the microphone. However, during treatment, the dental drill operates in the mouth of the patient and is thus only approximately 15 cm from the ears. Second, the drilling sound for artificial teeth was used as stimuli in the present experiment. The sound quality of drilling sounds may be more various (more metallic) in a clinical setting, because dentists use drills for the treatment of not only natural teeth, but also for the polishing or removal of various dental materials, such as hybrid plastics, porcelain, and metal alloys. Third, the length of each stimulus in the present experiment was considerably shorter than the typical duration of exposure during clinical treatment and was fixed based on consideration that the participants would be subjected to a total 120 s of sound stimuli in each session. Thus, patients may feel more uncomfortable when they are exposed to the dental drill sounds in the clinical setting. Finally, the sound stimuli in the present study were delivered through headphones; however, patients are able to detect both air-conducted and bone-conducted sound. For these reasons, the sound stimuli used in this study may not be fully representative of the sounds emitted by dental drills in a clinical setting.

## Conclusions

We conducted psychological measurements of the sound quality of dental drills using the semantic differential to facilitate potential modifications of the sounds of dental drills in order to create a more comfortable sound environment at dental clinics. Our analyses revealed that the impressions of dental drill sounds consisted of the factors “metallic and unpleasant” and “powerful”. Among the examined physical metrics, *L*_Aeq_ values were positively correlated with the “powerful impression”; calculated sharpness was related to the adjective scale values of the “metallic impression”; and both *L*_Aeq_ and sharpness predicted the “unpleasant impression” of the sound of dental drills. In addition, we found that *CI*, which is composed of *L*_Aeq_ and sharpness, appears to be a suitable index for evaluating the unpleasant feeling elicited by dental drills. These results demonstrate that both *L*_Aeq_ and sharpness influence the subjective impression of dental drill sounds. Our findings suggest that reducing the sound pressure level and refining the frequency characteristics of drilling sounds are important considerations for creating a comfortable sound environment in dental clinics. The results of our analyses also indicate that when evaluating the effects of dental drill sounds on the sound environment in dental clinics, it is not sufficient to measure the sounds of the drills under idling conditions alone. We are currently conducting further research regarding the relationship between the sound characteristics of dental drills and anxiety levels associated with dental treatment, as well as between the sound characteristics of dental drills and subjective impressions of such sounds when perceived via bone conduction.

## References

[1] Yamada T, Kuwano S, Ebisu S. A questionnaire survey on the effect of the sound of dental drills on the feeling of patients in dental clinics. Acoust Sci Tech. 2006;27: 305–308.

[2] Hearing conservation program, Occupational safety and health standards OSHA 1910.95(c). Occupational Noise Exposure. Occupational Safety & Health Administration, U.S. Department of Labor, Washington, USA, 1983.

[3] Wilson CE, Vaidyanathan TK, Cinotti WR, Cohen SM, Wangv SJ. Hearing-damage risk and communication interference in dental practice. J Dent Res. 1990;69: 489–493. 2407759 10.1177/00220345900690021401

[4] Sectos JC, Mahyuddin A. Noise levels encountered in dental clinical and laboratory practice. Int J Prosthodont. 1998;11: 150–157. 9709605

[5] International Organization for Standardization. ISO 14457: Dentistry—Handpieces and motors International Standard. Geneva, Switzerland, 2012.

[6] Japanese Industrial Standards Committee. JIS T 5906: Dental handpieces—Part 1: High-speed air-turbine handpiece. Japanese Industrial Standards Tokyo, Japan, 2001.

[7] Namba S. Sound quality In: Fastl H, Kuwano S, Schick A, editors. Recent trends in hearing research. Oldenburg: BIS; 1996 pp. 1–27.

[8] Fastl H. The psychoacoustics of sound-quality evaluation. Acustica. 1997;83(5): 754–764.

[9] Namba S, Kuwano S, editors. Method of psychological measurement for hearing research. Tokyo: Corona Publishing; 1998.

[10] Fastl H. Section 3: Psychoacoustics and product sound quality In: Kuwano S, editor. Recent topics in environmental psychoacoustics. Osaka: Osaka University Press; 2008 pp. 63–87.

[11] Namba S. Chapter 3: Design for industrial product sound quality In: Kuwano S, editor. Design on sound environment. Tokyo: Corona publishing; 2007 pp. 69–118.

[12] Kuwano S. On the design of product sound quality. J Acoust Soc Jpn. 2008;64: 551–555.

[13] Patsouras C, Fastl H, Widmann U, holzl G. Psychoacoustic evaluation of tonalcomponents in view of sound quality design for high-speed train interior noise. Acoust Sci Tech. 2001;23: 113–116.

[14] Barbot B, Lavandier C, Cheminée P. Perceptual representation of aircraft sounds. Appl Acoust. 2008;69(11): 1003–1016.

[15] Kuwano S, editor. Chapter 2: Design for auditory warning signals In: Design on sound environment. Tokyo: Corona publishing; 2007 pp. 37–68.

[16] Kuwano S, Namba S, Schick A, Hoege H, Fastl H, Thomas F, et al Subjective impression of auditory danger signals in different countries. Acoust Sci Tech. 2007;28(5): 360–362.

[17] Osgood CE. The nature and measurement of meaning. Psychol Bull. 1952;49: 197–237. 14930159 10.1037/h0055737

[18] Fastl H, Zwicker E, editors. Chapter 9: Sharpness and sensory pleasantness In: Psychoacoustics. Facts and models. Berlin Heidelberg: Springer; 2007 pp. 239–246.

[19] International Organization for Standardization. ISO 532: Acoustics–Method for calculating loudness level International Standard. Geneva, Switzerland, 1975.

[20] International Organization for Standardization. ISO 1996–1: Acoustics–Description, measurement and assessment of environmental noise–Part 1: Basic quantities and assessment procedures International Standard. Geneva, Switzerland, 2003.

[21] Von Bismarck G. Sharpness as an attribute of the timbre of steady state sounds. Acustica. 1974;30: 159–172.

[22] Terhardt E. On the perception of periodic sound fluctuations (roughness). Acustica. 1974;30: 201–213.

[23] Kuwano S, Namba S, Miura H. Advantages and disadvantages of A-weighted sound pressure level in relation to subjective impression of environmental noises. Noise Control Eng J. 1989;33: 107–115.

[24] Florentine M. Chapter 1: Loudness In: Florentine M, Popper A, Fay RR, editors. Perspectives on auditory research. New York: Springer handbook of auditory research; 2010 pp. 1–15.

[25] Namba S, Kuwano S. Psychological study on *L*eq as a measure of loudness of various kinds of noises. J Acoust Soc Jpn (E). 1984;5: 135–148.

[26] Namba S, Kuwano S, Kinoshita K, Kurakata K. Loudness and timbre of broad-band noise mixed with frequency modulated sounds. J Acoust Soc Jpn (E). 1992;13: 229–232.

[27] Kuwano S, Namba S, Kato T. Calculation of loudness level of time-varying sounds. In: Proceedings of the 39th International Congress and Exposition on Noise Control Engineering; [CD-ROM]. 2010 June 13–16; Lisbon, Portugal.

[28] Schlittenlacher J, Hashimoto T, Kuwano S, Namba S. Overall loudness of short time-varying sounds. In: Proceedings of the 43rd International Congress and Exposition on Noise Control Engineering; [USB]. 2014 Aug 23–26; Melbourne, Australia.

[29] Kuwano S, Namba S, Takehira O, Fastl H. Subjective impression of copy machine noises: An examination of physical metrics for the evaluation of sound quality. In: Proceedings of the 38th International Congress and Exposition on Noise Control Engineering; [CD-ROM]. 2009 Aug 23–26; Ottawa, Canada.

[30] Thurston LL. Multiple factor analysis—a development and expansion of the Vectors of Mind. Am J Psychol. 1948;61(1): 129–131.

[31] Japanese Industrial Standards Committee. JIS Z 8731: Acoustics—Description and measurement of environmental noise Japanese Industrial Standards. Tokyo, Japan 1999.

